# Antibiotic Resistance of *Enterococcus* spp. Isolated from the Urine of Patients Hospitalized in the University Hospital in North-Central Poland, 2016–2021

**DOI:** 10.3390/antibiotics11121749

**Published:** 2022-12-03

**Authors:** Zuzanna Kraszewska, Krzysztof Skowron, Joanna Kwiecińska-Piróg, Katarzyna Grudlewska-Buda, Jana Przekwas, Natalia Wiktorczyk-Kapischke, Ewa Wałecka-Zacharska, Eugenia Gospodarek-Komkowska

**Affiliations:** 1 Department of Microbiology, Nicolaus Copernicus University in Toruń, L. Rydygier Collegium Medicum in Bydgoszcz, 9 M. Skłodowska-Curie St., 85-094 Bydgoszcz, Poland; 2 Department of Food Hygiene and Consumer Health, Wrocław University of Environmental and Life Sciences, 50-375 Wrocław, Poland

**Keywords:** urinary tract infections, *Enterococcus*, empirical therapy, urology, antimicrobial susceptibility

## Abstract

Urinary Tract Infections (UTIs) are common outpatient and inpatient infections, often treated with empirical therapy. *Enterococcus* spp. is responsible for about 10% of UTIs. This study aimed to determine the necessity of changing the empirical treatment of UTIs caused by *Enterococcus* spp. The evaluation was performed for 542 *Enterococcus* strains isolated from urine samples in the years 2016–2021. We identified three *Enterococcus* species that were found: *E. faecalis* (389, 71.8%), *E. faecium* (151, 27.8%) and *E. gallinarum* (2, 0.4%). *E. faecalis* was the dominant species every year. Among *E. faecalis,* the most prevalent was resistance to norfloxacin (51.4%). Almost all *E. faecium* strains (150, 99.3%) were resistant to beta-lactams and norfloxacin. Eighty-three strains (55.0%) were resistant to vancomycin and 72 (47.7%) to teicoplanin. *E. faecium* strains showed a significantly higher percentage of resistance mechanisms GRE (Glicopeptide-Resistant *Enterococcus*) (72, 48.7%) and VRE (Vancomycin-Resistant *Enterococcus*) (11, 7.3%), while only five strains of *E. feacalis* showed a VRE mechanism (1.3%). In the therapy of *E. faecalis* UTIs, ampicillin and imipenem still remain effective. However, the above-mentioned antibiotics, as well as fluoroquinolones, are not recommended in the treatment of UTIs of *E. faecium* etiology.

## 1. Introduction

The presence of a significant amount of microorganisms in urine may lead to Asymptomatic Bacteriuria (ABU) or indicate Urinary Tract Infections (UTIs), including uncomplicated and complicated forms. UTIs may affect only the lower urinary tract and occur as cystitis (bladder infection), causing frequent urination, lower abdominal pain and a feeling of pressure or burning in the urethra. When UTIs affect the upper urinary tract, such as ureters and kidneys, they may also cause fever, vomiting, back pain and even hematuria [1]. Rarely, UTIs may lead to renal failure or urosepsis and be life-threatening. UTIs account for 10–20% of adult infections and approximately 50% of nosocomial infections (due to frequent and long-term catheterization) [2]. Furthermore, urologic surgeries may increase the risk of UTIs [3]. The bacteria enter the urinary tract by the bloodstream or the lymphatic route, but the most common way of infection is the ascending route directly through the urethra. The difference in the length of male and female urethra explains why women are more exposed to UTIs [4]. Uropathogens are a diverse group of microorganisms, mainly Gram-negative rods, such as *Escherichia coli* (more than 80% of infections), *Proteus mirabilis* or *Klebsiella pneumoniae* [5]. Another group is Gram-positive cocci such as *Staphylococcus saprophyticus* or *Enterococcus* spp., responsible for less than 10% of nosocomial UTIs [5,6]. In recent years, the number of fungal UTIs has also increased, especially in children, due to immunosuppression and antimicrobial therapy [7].

According to the 2020 National Antibiotic Protection Program recommendations published by National Medicine Institute in Poland [8], uncomplicated UTIs in women should be empirically treated with cotrimoxazole (which is also indicated in the treatment and prevention of recurrent UTIs), nitrofurantoin or fosfomycin. The second choice treatment is amoxicillin with clavulanic acid and fluoroquinolones, used as a last resort in the case of uncomplicated forms of UTIs. In contrast, fluoroquinolones are the first choice treatment in men with UTIs and acute pyelonephritis. UTIs of enterococcal etiology are treated with ampicillin or amoxicillin. For *E. faecium* and *E. faecalis*, cotrimoxazole can also be used. Cotrimoxazole or amoxicillin with clavulanic acid treatment should be preceded by a single dose of a long-acting antibiotic, such as ceftriaxone. In the case of resistance to penicillins and cotrimoxazole, ciprofloxacin is recommended. The therapy in the case of Urinary Tract Infection according to Polish national guidelines does not differ significantly from European guidelines. The European Association of Urology Guideline for Urological Infection treatment, published in 2020, recommends fluorochinolones or cephalosporins for uncomplicated UTIs. In the case of complicated or community-acquired UTIs, the first choice treatment is amoxicillin in combination with aminoglicosides. The second choice is fluorochinolones if the ciprofloxacin resistance percentage threshold in the local area is less than 10% [9]. Furthermore, Kang et al. suggest the choice of fluoroquinolones, co-trimoxazole and cephalosporins in the empirical treatment of UTI, additionally indicating that empirical therapy should be selected according to local guidelines and on the basis of antibiotic susceptibility data [10].

In recent years, resistance to aminopenicillins, imipenem and fluoroquinolones in eneterococci (especially in *E. faecium*) has been increasing, leaving poor therapeutic options for UTI treatment. This may explain the necessity of glycopeptide antibiotics use, such as vancomycin and teicoplanin [11]. Among MDR enterococci, three dangerous resistance mechanisms are spreading: VRE (Vancomycin-Resistant *Enterococcus*), GRE (Glycopeptide-Resistant *Enterococcus*), and even LRE (Linezolid-Resistant *Enterococcus*) [12]. The use of glycopeptides and linezolid in empirical therapy may increase enterococcal resistance to these antibiotics and lead to the spread of super-resistant bacteria with no chance of treatment.

This study aimed to determine the prevalence of *Enterococcus* spp. as uropathogens in hospitalized patients between 2016–2021. The second goal was to retrospectively assess the resistance of selected strains to antibiotics empirically used in UTIs of enterococcal etiology in hospitals. Such a comparison allowed us to verify the necessity of the treatment model modification due to the increased antibiotic resistance.

## 2. Results

In this study, 542 *Enterococcus* spp. isolates collected from the urine of patients suffering from uncomplicated and complicated UTIs were retrospectively evaluated.

### 2.1. Species Prevalence Analysis

The analysis showed an increase in the number of enterococci isolations from urine samples over years 2016–2021. We have found three *Enterococcus* species in positive urine samples: 389 (71.8%) were *E. faecalis*, 151 (27.9%) were *E. faecium* and 2 (0.4%) of them were *E. gallinarum*. *E. faecalis* was the dominant species every year. Table 1 presents the number of isolates and their species distribution. From all 542 *Enterococcus* spp. isolates, 118 (21.8%) were re-isolated from the same patients (Table 1). Due to the observed differences in the antibiogram and the time intervals between isolations (more than two weeks), re-isolates were included in the evaluation and treated like different strains. Differences in the prevalence of the two most frequently isolated species between particular years were mostly insignificant (*p* > 0.05). The exception was in 2020, when we noticed highly significant differences in the prevalence of *E. faecium* and *E. faecalis*, in comparison to 2017 (*p* = 0.0007) and 2019 (*p* = 0.0008), as well as a significant difference compared to 2018 (*p* = 0.09). In addition, significant differences in the above-mentioned parameters were also noticed when comparing 2021 with 2017 (*p* = 0.006) and 2019 (*p* = 0.009).

A higher percentage of enterococcal strains was found in patients of the Urology Clinic (52.0%) of which 67.7% were isolated from men. Among isolates found in patients of the Nephrology Clinic (48.0%), isolates collected from men were also obtained more often (53.9%). Table 2 presents the number of isolated strains from man and women in two assessed clinics and their species distribution. Statistical analysis did not reveal significant differences in the occurrence of *E. faecalis* and *E. faecium* in men and women (*p* = 0.011).

### 2.2. Antimicrobial Susceptibility Testing

*E. faecium* showed a higher level of resistance to all antibiotics used in the study than *E faecalis* (*p* ≤ 0.001), with the exception of linezolid (*p* = 0.533). Among *E. faecalis,* most prevalent was resistance to norfloxacin (51.4%). Only two (0.5%) isolates additionally showed resistance to the beta-lactams and 5 (1.29%) were resistant to vancomycin. On the other hand, almost all *E. faecium* strains (99.3%) were resistant to beta-lactams and norfloxacin. Eighty-three (55.0%) of them were resistant to vancomycin and 47.7% to teicoplanin. Both *E. gallinarum* strains (100.0%), which are naturally vancomycin-resistant, were resistant to norfloxacin. One *E. faecalis* strain, isolated in 2020, was resistant to linezolid and norfloxacin but sensitive to beta-lactams and glycopeptides. Table 3 illustrates the antimicrobial resistance of *E. faecalis* and *E. faecium* strains collected between 2016–2020.

### 2.3. Occurrence of Resistance Mechanisms and Resistance Patterns

Among all assessed enterococcal isolates, three resistance mechanisms were observed, as shown in Table 4. There were significantly higher percentages of GRE (48.7%) (*p* < 0.00001) and VRE (7.3%) (*p* = 0.002) *E. faecium*, while only 5 (1.3%) strains of *E. feacalis* were VRE. Table 5 presents the Multi-Drug Resistance (MDR) pattern of the tested strains.

## 3. Discussion

UTIs most often affect children <10 years old and adults older than 60, mainly due to more frequent anatomical defects in the urinary tract and long-term catheterization [13,14]. In our study, more than half (51.7%) of strains were isolated from midstream urine. A high percentage of isolation from this type of material may be due to improper preparation of the patient for this procedure (insufficient hygiene or a sample taken from the first urine stream). As much as 36.2% of the isolates were found in urine collected through the catheter. Many studies indicate the ability of *Enterococcus* spp. to form a biofilm structure on the catheter surface, which may be the cause of this phenomenon [15,16]. Moreover, our results indicate more frequent isolation of *E. faecium* (39.8%) from urine collected through the catheter than from other urine samples (18.2—21.2%). It may suggest an association with biofilm formation by *E. faecium* on the catheter surface. More likely, it explains that catheter is applied in people in advanced stages of the disease, often infected with more invasive and antibiotic-resistant strains.

A decade ago, the presence of genes responsible for adhesion and biofilm formation has been rarely observed in the genome of *E. faecium* than *E. faecalis* [17,18]. However, recent publications indicate that the percentage of biofilm-forming *E. faecium* has been increasing [19,20]. Scientists have proved that in hospitals, antibiotics administered to patients, both in targeted and empirical therapy, often reach sub-inhibitory concentrations and enhance biofilm formation by enterococci [21].

In our study, we showed that in the years 2016–2021, the number of UTI cases with enterococcal etiology in Urology and Nephrology Clinics, increased year by year. Comparing data from the 2015 and 2019 European Centre for Disease Prevention and Control (ECDC) reports on Intensive Care Unit (ICU) infections, a similar trend can be seen in most of the analyzed European countries [22,23]. However, in a British report, analyzing urine samples collected in 2005–2014, Toner et al. showed a systematic decrease in the number of *Enterococcus* spp. strains [24]. In 2012, in European countries, *Enterococcus* spp. was the third bacterium most frequently isolated from reported nosocomial UTIs, right after *Escherichia coli* and *Candida* spp. In 2017, these bacteria were already in second place [22,23]. In some countries (Luxemburg, Slovakia), according to the 2019 ECDC report, enterococci have even become the main etiological agent of ICU-acquired UTIs [23]. A retrospective analysis of UTI incidents in 2006–2016 performed by Suh et al. shows that also among patients below 19 years old enterococci are the second most frequent etiological factor, accounting for 6.7% of all isolates [25]. In contrast, Ganesh et al. have reported on only 0.5% of *Enterococcus* spp.-positive urine samples collected from infants and children in Nepal [26]. Bitsori et al. concluded that enterococci as an etiologic factor of UTIs in children were more often associated with urinary tract anatomical abnormalities and worse prognosis in corrective surgeries than Gram-negative bacteria [27].

According to our results, of the three species of enterococci isolated from the analyzed urine samples, the most common was *E. faecalis* (71,8%). Other studies confirm that *E. faecalis* is a more common etiological factor of UTIs than *E. faecium* [24,28,29,30,31].

Although UTIs are generally more common in women than in men, in our study men more often (61.1%) suffered from UTIs of *Enterococcus* spp. etiology. Similar results were obtained by Barros et al. in Brazil, where male patients with enterococcal UTIs accounted for 57.0% [14]. Such results are probably due to the specificity of the clinics, where men are principally hospitalized. Interestingly, Cornia et al., studying bacteriuria in a large group of older men, noticed that enterococci were most often (24.3%) isolated species from UTI cases [32]. This might be associated with any anatomical defects and abnormalities and the higher frequency of surgical procedures in the urinary tract in men.

In our study, we assessed the susceptibility of enterococci to representatives of various antibiotics groups used in UTIs treatment, including aminopenicillins (ampicillin), carbapenems (imipenem), fluoroquinolones (norfloxacin), glycopeptides (vancomycin and teicoplanin) and oxazolidinones (linezolid).

Fluorochinolones are one of the recommended antimicrobial groups in empirical UTI treatment for adults. The use of these antibiotics in children is not recommended due to their toxic effect on chondrocytes, which was proven in studies on juvenile animals [33]. In our study, we used the norfloxacin disc as the screening method for fluoroquinolones susceptibility testing. The examined *Enterococcus* spp. isolates showed the highest resistance to norfloxacin (65.0%) including 51.44% *E. faecalis* and nearly all of *E. faecium* (99.3). In other studies, scientists also demonstrated a higher level of fluoroquinolones-resistant *E. faecium* (>80%) than *E. faecalis* (>50%) [34,35,36,37]. In addition, there are works confirming the growing resistance to fluoroquinolones in non-hospital isolated enterococci, e.g., from dental or food examinations [38,39].

Enterococci are characterized by intrinsic resistance to some beta-lactam antibiotics (cephalosporins, meropenem), which is related to the lack of appropriate Penicillin Binding Proteins (PBP) in their structure. Furthermore, *Enterococcus* spp. may overproduce modified PBP5 proteins having low affinity for beta-lactams and conferring resistance to ampicillin and amoxicillin [40]. The results of our work confirmed that the phenomenon of beta-lactam resistance is more frequent in *E. faecium* than *E. faecalis*. Nearly all tested *E. faecium* strains (99.3%) were resistant to ampicillin and imipenem, while only two isolates of *E. faecalis* (0.5%) showed that resistance pattern. Protonotarious et al. published similar results, where over the years 2002–2007, the ampicillin-resistant *E. faecalis* ranged between 0.4–2.3%, and in the case of *E. faecium* strains, resistance to ampicillin was much higher and amounted to 76.5–89.9% [41]. An interesting relationship was observed by Rathnayake et al., who investigated antibiotic resistance in enterococci isolated from clinical materials and from water. The researchers showed that in *E. faecium* ampicillin resistance was significantly higher among clinical isolates (72.7%) than among water isolates (27.3%), probably due to the frequent contact of clinical isolates with antibiotics used in hospital treatment.

*Enterococcus* spp. resistance to glycopeptides is due to the presence of several genes belonging to the van gene complex. The products of those genes modify the side chain in the peptidoglycan molecules and limit or even prevent the structure from binding to the antibiotic [42]. It allows the bacterial cell to continue the cell wall synthesis despite the presence of the glycopeptides. In this study, *E. faecium* showed greater resistance to both glycopeptides: vancomycin (55.0%) and teicoplanin (47.68%). On the other hand, the percentage of strains resistant only to vancomycin was low (3.0%). We did not notice any teicoplanin-resistant and vancomycin-sensitive strains at the same time. Both (100.0%) *E. gallinarum* isolates were resistant to vancomycin, probably due to an intrinsic low-level vancomycin resistance for this species [43]. Zhanel et al. described a large group of enterococci isolated from urine and showed that 40.1% of *E. faecalis* and 93.6% of *E. faecium* were resistant to vancomycin. They also showed that approximately 75.0% of all vancomycin-resistant isolates were resistant to teicoplanin, confirming the statistics obtained in our study [44]. We did not notice an increasing trend in the occurrence of glycopeptide resistance mechanisms in 2016–2021. Different results were obtained in other studies [45,46]. Ayobami et al., analyzing the antibiotic susceptibility of enterococci isolated from the bloodstream in Europe (hospital clinics and the emergency department), observed an increasing number of VRE isolates over 2012–2018. Moreover, they showed a significantly higher percentage of VRE strains isolation in hospitalized patients than in outpatients, which indicates the hospital is an environment for spreading infections with multi-drug resistant *Enterococcus* strains [47].

Linezolid resistance in *Enterococcus* spp. results from point mutations in the 23S rRNA gene that changes ribosomal proteins L3, L4 and L22. These changes contribute to the production of altered ribosomal RNA, preventing the proper action of the antibiotic. There are also genes responsible for the linezolid-resistance mechanism, described first in *E. faecium*, such as antibiotic resistance genes (cfr) and newly identified efflux-pump genes (optrA, poxtA) [48,49]. In our work, only one *E. faecalis* isolate from 2020 showed resistance to linezolid, while being sensitive to beta-lactams, imipenem and glycopeptides. Burleson et al. analyzed three LRE strains and showed that all of them were sensitive to ampicillin and one additionally to norfloxacin, vancomycin and teicoplanin, showing that resistance to linezolid is not associated with resistance to other antimicrobial groups [50]. Wardenburg et al. showed that enterococcal strains from different geographic regions display different resistant strategies to linezolid. This is associated with increased administration as a last resort treatment for multi-drug resistant *Enterococcus* spp. strains in hospitals [49].

The phenomenon of ampicillin resistance among hospital strains of *E. faecium* may be related to the presence of a genetic subpopulation called clonal complex 17 (CC17) [51]. Such strains also often contain a pathogenicity island and the *esp* virulence gene responsible for biofilm formation [52]. Studies have shown that *E. faecium* strains of the CC17 subpopulation are often also resistant to glycopeptides, fluoroquinolones, and even to linezolid [52,53,54]. This may explain the results of our study on the high level of antibiotic resistance among *E. faecium* isolates.

Our research confirmed that *E. faecalis* is the most often isolated species from enterococcal UTIs. *E. faecium* also frequently contributes to UTIs, while other enterococcal species cause infections sporadically. The obtained results indicate more frequent isolation of enterococci from men, probably due to the specificity of the analyzed clinics and more frequent urological procedures performed in the male urinary tract. Antibiotic susceptibility analysis of enterococci strains, evaluated in this study, demonstrated a very high level of resistance to norfloxacin—more than half of *E. faecalis* and almost all *E. faecium* strains showed resistance in the screening test. The use of fluoroquinolones in UTI treatment should therefore be limited when other therapeutic options exist. The results of the retrospective analysis also showed a high level of resistance to glycopeptides. Moreover, almost all strains resistant to vancomycin were also resistant to teicoplanin, which is probably the result of a poor treatment model. Administration of teicoplanin to a patient, with concomitant vancomycin resistance, should be conscientiously monitored and only used when necessary. The results of our study also confirmed that a significant proportion of *Enterococcus* spp. strains, including all *E. faecium*, isolated from urine are resistant to at least three groups of antibiotics. Although urinary isolated enterococci are rarely resistant to linezolid and we did not observe the presence of Extensively-Drug Resistant (XDR) strains. Linezolid was the only treatment option in many of the observed cases.

In conclusion, for assessed clinics, beta-lactams should be still the first choice in the treatment of UTIs of *E. faecalis* etiology. Imipenem can be administered empirically at an increased dose due to the risk of the strain acquiring resistance to carbapenems. The high level of *E. faecalis* strains resistant to norfloxacin suggests that fluoroquinolones are not suitable for empirical therapy in the clinics under evaluation. However, the susceptibility to other antibiotics should be determined before drawing such a conclusion. In the case of UTIs with *E. faecium* etiology, the use of beta-lactams or fluoroquinolones in empirical therapy is not recommended in the clinics under evaluation, as this may result in treatment failure. Since all *E. faecium* strains were MDR strains, the administration of glycopeptides was inevitable. The results of our research led to the conclusion that in the evaluated hospital clinics, empirical treatment in the case of enterococcal UTI should be adjusted to the species, if possible, and carefully monitored. Although current empiric therapy for UTIs of unknown etiology is usually effective, our research shows that fluoroquinolones should not be used when *Enterococcus* spp. Is the possible etiological factor.

## 4. Materials and Methods

**Figure 1:**
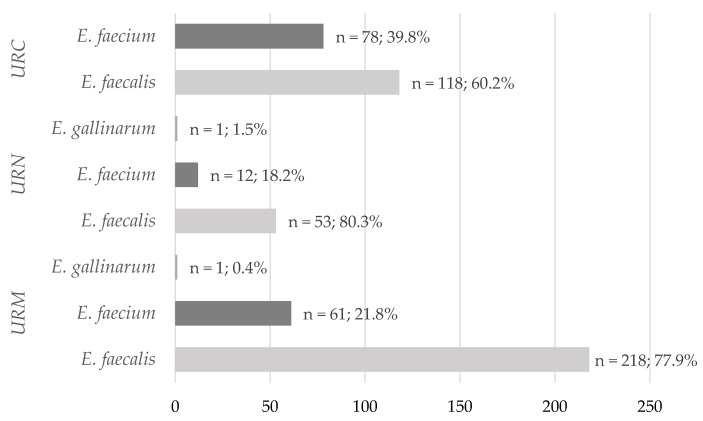
Percentage of *E. faecalis* and *E. faecium* strains isolated from different types of urine samples in Nephrology and Urology clinics over the years 2016–2021 (n = 542). URC—urine samples collected through the catheter, URM—midstream urine samples, URN—urine samples collected from a nephrostomy.

All patients of the study group were adults, aged between 20 and 101 years, hospitalized in Nephrology and Urology Clinics at the Dr. Antoni Jurasz University Hospital No. 1 in Bydgoszcz between 2016–2021. Urine samples were collected as a result of routine diagnostic procedures at the request of physicians in charge of the patients. There were three different ways of collecting urine samples for the study: midstream urine samples (51.7%), urine collected from a nephrostomy (12.2%) or through the catheter (36.2%). Of each type of urine sample, *E. faecalis* (60.2–80.3%) was the most frequently isolated (Figure 1). In patients included in this study, UTIs were recognized on the basis of clinical symptoms (fever, feeling of pressure or burning in the urethra, dysuria and/or lower abdominal pain and/or hematuria) and confirmed by microbiological tests (the presence of enterococci in the urine ranged from >10^3^ to ≥10^5^ colony forming units per milliliter (CFU/mL) depending of the type of urine samples collection).

### 4.1. Identification of Enterococcal Uropathogenes

Identification of isolated *Enterococcus* spp. strains was performed using the MALDI BioTyper Microflex mass spectrometer (Bruker, Billerica, MA, USA), which uses the MALDI-TOF MS (Matrix Assisted Laser Desorption Laser–Time of Flight Mass Spectrometry), following the manufacturer’s recommendations. The probability of assigning a microorganism to an appropriate species was determined on the basis of the parameter described in the system as score value, the value of which was assessed in accordance with the manufacturer’s instructions (score value > 2.000 means the highly probable assignment of the microorganism to the species).

### 4.2. Antimicrobial Susceptibility Testing

Antimicrobial susceptibility of all 542 *Enterococcus* spp. was determined using the Kirby–Bauer disc diffusion method on Mueller Hinton II Agar (MHA, Becton Dickinson, Franklin Lakes, NJ, USA) according to the EUCAST (European Committee on Antimicrobial Susceptibility Testing) disc diffusion method ver. 9.0 recommendations [55]. The following antibiotic discs were used: ampicillin (2 μg), imipenem (10 μg), norfloxacin (10 μg), vancomycin (5 μg), teicoplanin (30 μg) and linezolid (10 µg) (OXOID, Basingstoke, UK). After 16–18 h of incubation at 35 °C, under aerobic conditions, inhibition zones around the discs were measured. The results were interpreted according to the EUCAST ver. 11.0 breakpoint tables [56]. The study included reference strain *E. faecalis* ATCC^®^ 29212™ from the American Type Culture Collection (ATCC^®^).

The presence of resistance mechanisms was assessed using the disc diffusion method, determining the resistance to selected antibiotics, according to the EUCAST ver. 11.0 breakpoint tables [56]. The assessed isolates were considered GRE if they showed resistance to both glycopeptide antibiotics used in the study (vancomycin and teicoplanin), while the strains showing resistance to vancomycin with simultaneous sensitivity to teicoplanin were considered VRE. Enterococcal strains resistant to linezolid were considered LRE.

### 4.3. Data Analysis

The Chi-squared test calculated in Statistix 13.0 (TIBCO Software, Palo Alto, CA, USA) was used to determine the significant differences in the prevalence of isolates in particular years, different clinics, gender and also in the occurrence of antibiotic resistance. Differences of *p* ≤ 0.01 were considered significant, while *p* ≤ 0.001 was highly significant.

## Figures and Tables

**Table 1:** The prevalence of enterococcal strains isolated from the urine of patients hospitalized in Nephrology and Urology Clinics between 2016–2021 and their species distribution.

Year		Enterococcal Species	n	%	Re-Isolates, n (%)
2016 (n = 36)	**Occurrence of Uropathogens**	*E. faecalis*	26	72.2	4 (11.1)
		*E. faecium*	10	27.8	
2017 (n = 47)		*E. faecalis*	26	55.3	11 (23.4)
		*E. faecium*	20	42.6	
		*E. gallinarum*	1	2.1	
2018 (n = 58)		*E. faecalis*	37	63.8	13 (22.4)
		*E. faecium*	21	36.2	
2019 (n = 100)		*E. faecalis*	62	62.0	24 (24.0)
		*E. faecium*	38	38.0	
2020 (n = 167)		*E. faecalis*	134	80.2	44 (26.3)
		*E. faecium*	32	19.2	
		*E. gallinarum*	1	0.6	
2021 (n = 134)		*E. faecalis*	104	77.6	22 (16.4)
		*E. faecium*	30	22.4	

**Table 2:** The prevalence of enterococcal strains isolated from the urine of patients hospitalized in Nephrology and Urology clinics over the years 2016–2021 and their species distribution in different gender groups (n = 542).

Uropathogens	Hospital Clinics			
	Nephrology (n = 260)		Urology (n = 282)	
	Women (n = 120)	Men (n = 140)	Women (n = 91)	Men (n = 191)
	Occurrence of Uropathogens, n (%)			
*E. faecalis*	64 (53.3)	90 (64.3)	75 (82.4)	160 (83.8)
*E. faecium*	56 (46.7)	49 (35.0)	16 (17.6)	30 (15.7)
*E. gallinarum*	0	1 (0.7)	0	1 (0.5)

**Table 3:** Percentage of antimicrobial resistance of *E. faecalis* and *E. faecium* strains isolated from the urine of patients hospitalized in Nephrology and Urology Clinics and their species distribution divided into years 2016–2021.

Number of All Isolates			AMP		IPM		NOR		VAN		TEC		LZD	
			n	(%)	n	(%)	n	(%)	n	(%)	n	(%)	n	(%)
2016	*E.faecalis*	26	0	(0)	0	(0)	16	(61.5)	0	(0)	0	(0)	0	(0)
	*E. faecium*	10	10	(100.0)	10	(100.0)	10	(100.0)	2	(2.0)	2	(2.0)	0	(0)
2017	*E.faecalis*	26	0	(0)	0	(0)	16	(61.5)	1	(3.9)	0	(0)	0	(0)
	*E. faecium*	20	19	(95.0)	19	(95.0)	20	(100.0)	12	(60.0)	11	(55.0)	0	(0)
2018	*E.faecalis*	37	0	(0)	0	(0)	19	(51.4)	0	(0)	0	(0)	0	(0)
	*E. faecium*	21	21	(100.0)	21	(100.0)	21	(100.0)	14	(66.7)	14	(66.7)	0	(0)
2019	*E.faecalis*	62	0	(0)	0	(0)	34	(54.8)	0	(0)	0	(0)	0	(0)
	*E. faecium*	38	38	(100.0)	38	(100.0)	37	(100.0)	23	(60.5)	23	(60.5)	0	(0)
2020	*E.faecalis*	134	0	(0)	0	(0)	64	(47.8)	1	(0.8)	0	(0)	1	(0.8)
	*E. faecium*	32	32	(100.0)	32	(100.0)	32	(100.0)	14	(43.8)	11	(34.4)	0	(0)
2021	*E.faecalis*	104	2	(1.9)	2	(1.9)	57	(54.8)	3	(2.9)	0	(0)	0	(0)
	*E. faecium*	30	30	(100.0)	30	(100.0)	30	(100.0)	18	(60.0)	11	(36.7)	0	(0)
Total	*E.faecalis*	389 (A)	2	(0.5)	2	(0.5)	200	(51.4)	5	(1.3)	0	(0)	1	(0.3)
	*E. faecium*	151 (B)	150	(99.3)	150	(99.3)	150	(99.3)	83	(55.0)	72	(47.7)	0	(0)
*p*-Value Comparison of A and B			<0.00001		<0.00001		<0.00001		<0.00001		<0.00001		0.533	

AMP—ampicillin, IPM—imipenem, NOR—norfloxacin, VAN—vancomycin, TEC—teicoplanin, LZD—linezolid; *p*-Value (results of Chi-square test).

**Table 4:** Occurrence of resistance mechanism (VRE, GRE and LRE) in enterococcal strains isolated from the urine of patients hospitalized in Nephrology and Urology Clinics between 2016–2021.

Antimicrobial Resistant Mechanism	*E. faecalis* (n = 389)	*E. faecium* (n = 151)	*p* = Value
	Isolates, n (%)		
VRE	5 (−1.3)	11 (7.3)	0.0002
GRE	0	72 (42.7)	<0.00001
LRE	1 (0.3)	0	0.533

VRE—Vancomycin-Resistant *Enterococcus*, GRE—Glicopeptide-Resistant *Enterococcus*), LRE—Linezolid-Resistant *Enterococcus*; *p*-Value (results of Chi-square test).

**Table 5:** Multi-drug resistance patterns of enterococcal strains isolated from the urine of patients hospitalized in Nephrology and Urology Clinics between 2016–2021 (n = 542).

Uropatogenes	Combination of Antibiotics	No. of Isolates	
		n	%
*E. faecalis* (n = 389)	AMP/AMC, IPM, NOR	1	0.3
	AMP/AMC, IPM, NOR, VAN	1	0.3
*E. faecium* (n = 151)	AMP/AMC, IPM, NOR	68	45.0
	AMP/AMC, IPM, NOR, VAN	11	7.3
	AMP/AMC, IPM, NOR, VAN, TEC	72	47.7

AMP—ampicillin, IPM—imipenem, NOR—norfloxacin, VAN—vancomycin, TEC—teicoplanin.

## Data Availability

Not applicable.
